# Episignature-based modelled first-tier diagnostic approach in the Dutch Caribbean: advancing equal care through epigenetic classification

**DOI:** 10.3389/fgene.2026.1803993

**Published:** 2026-05-20

**Authors:** Liselot van der Laan, Amanda Luijckx, Shirley Lo-A-Njoe, Ginette M. Ecury-Goossen, Farah A. Falix, Sonja Faries, Meindert E. Manshande, Patricia A. E. A. C. Philippi, Evelyn M. van der Plas, Hans D. Veenhuis, Louise Rafael-Croes, Marcel M. A. M. Mannens, Bekim Sadikovic, Peter Henneman, Mariëlle Alders, Mieke M. van Haelst

**Affiliations:** 1 Department of Human Genetics, Amsterdam UMC, Amsterdam, Netherlands; 2 Amsterdam Reproduction and Development Research Institute, Amsterdam University Medical Center (AUMC), University of Amsterdam, Amsterdam, Netherlands; 3 Emma Center for Personalized Medicine, Amsterdam UMC, Amsterdam, Netherlands; 4 Department of Pediatrics, Curaçao Medical Center (CMC), Willemstad, Curaçao; 5 Department of Pediatrics, Dr. Horacio E. Oduber Hospital, Oranjestad, Aruba; 6 Verspeeten Clinical Genome Centre, London Health Sciences Centre, London, ON, Canada; 7 Department of Pathology and Laboratory Medicine, Western University, London, ON, Canada

**Keywords:** Dutch Caribbean, episignatures, equal healthcare, genetic services, healthcare challenges, rare disorders

## Abstract

**Introduction:**

Access to advanced genetic testing in the Dutch Caribbean is limited due to financial and logistical constraints. As a result, many patients with rare disorders remain undiagnosed or receive uncertain results, leading to unequal genetic care. DNA methylation profiles, or episignatures, provide sensitive and specific biomarkers. The clinically validated EpiSign™ assay offers a cost-effective and rapid diagnostic approach.

**Methods:**

In this study, we evaluated EpiSign™ as a modelled first-tier screening approach within a proposed diagnostic workflow in a resource-limited setting and assessed its diagnostic yield and clinical utility in 19 patients from the Dutch Caribbean. Peripheral blood DNA was analyzed using the EPIC array, and methylation profiles were classified via a support vector machine algorithm against the EpiSign™ Knowledge Database. All patients underwent ID-WES and/or CGH-array analysis as part of the diagnostic workup. Positive EpiSign™ findings were subsequently confirmed by these molecular analyses.

**Results:**

EpiSign™ helped provide a clinical diagnosis for six cases, including Fragile X syndrome, Intellectual Developmental Disorder X-linked 93 (MRX93), ReNU syndrome, and Kleefstra syndrome 1, all confirmed by subsequent analyses. Several patients harbored variants of uncertain significance in genes not yet associated with known episignatures.

**Discussion:**

These results demonstrate that EpiSign™ provides actionable diagnostic insights as a first-tier diagnostic tool, particularly when combined with genomic analyses in the absence of parental samples. Its dynamic database allows reanalysis as new episignatures are identified, increasing its potential clinical utility in resource-limited settings.

## Introduction

The Dutch Caribbean, part of the Kingdom of the Netherlands, comprises six islands: Aruba, Bonaire, Curaçao, St. Maarten, St. Eustatius, and Saba. These islands vary in size, population, and healthcare infrastructure, and their geographic location limits access to specialized medical services. Aruba, Curaçao, and St. Maarten host larger medical centers with some (sub)specialist care, whereas smaller islands such as Bonaire, St. Eustatius, and Saba rely heavily on medical transfers to neighboring islands or the mainland.

Genetic services are limited, with biannual visits from Dutch clinical geneticists and restricted access to advanced diagnostics such as next-generation sequencing (NGS) (Verberne et al., 2021). Consequently, many patients with genetic (neurodevelopmental) remain undiagnosed or receive uncertain results due to limited access to comprehensive genomic testing, including trio-based sequencing and follow-up analyses, delaying appropriate care and affecting family wellbeing (Verberne et al., 2022a).

DNA methylation profiles, or episignatures, provide a sensitive and specific biomarker for the classification of variants of uncertain significance (VUS). The clinically validated EpiSign™ assay utilizes these methylation patterns to identify rare disorders, and its continuously expanding database allows reanalysis of previously tested samples as new episignatures are discovered. Compared with conventional genetic testing, EpiSign™ offers a standardized approach for diagnostic screening of neurodevelopmental disorders and enables interpretation of variants of uncertain significance (VUS) in a growing number of episignature disorders. These features make it particularly suitable as a first-tier diagnostic tool in resource-limited settings (Aref-Eshghi et al., 2020; Levy et al., 2022; Sadikovic et al., 2019; Kerkhof et al., 2024). However, episignature testing is not applicable in prenatal and neonatal settings due to dynamic epigenetic changes during early development.

In this study, we evaluated EpiSign™ as a modelled first-tier screening tool to streamline the diagnostic workflow and potentially reduce the need for sequential diagnostic testing in resource limited settings, in 19 patients from the Dutch Caribbean, assessing both its diagnostic yield and practical utility in a setting with limited access to advanced genetic diagnostics.

## Methods

### Patient cohort

We included 19 patients from the Dutch Caribbean (13 males and 6 females) in this study between 2023 and 2025. All patients underwent standard diagnostic testing (Intellectual disability-whole-exome sequencing (ID-WES) and/or CGH-array), and EpiSign™ was applied in parallel. For analytical purposes, we modelled a first-tier EpiSign™ approach to evaluate its potential diagnostic yield and its impact on subsequent molecular testing compared with the standard diagnostic workflow.

### DNA extraction and methylation profiling

Peripheral blood samples were collected from all patients, and DNA was extracted using standard protocols. DNA methylation was assessed using the Illumina Infinium MethylationEPIC BeadChip (EPIC v2.0) array (Illumina, San Diego, CA, United States). Raw intensity data (IDAT files) were imported into R for preprocessing, including standardization, background correction, and filtering. Beta values, ranging from 0 (no methylation) to 1 (complete methylation), were calculated for downstream analysis (Levy et al., 2022).

### EpiSign™ assay and classification

Methylation data were analyzed using the clinically validated EpiSign™ assay, as described previously (Levy et al., 2022). A complete list of disorders included in the EpiSign™ v5.1 panel is available at: https://genoomdiagnostiek.nl/wp-content/uploads/EpiSgnv5-menu_v5.1.pdf. Processed beta values were input into a support vector machine (SVM) classifier specifically trained for EpiSign™ disorders. The classifier utilizes the EpiSign™ Knowledge Database, which contains methylation profiles from reference disorder-specific cohorts and unaffected controls. Disorder-specific Methylation Variant Pathogenicity (MVP) scores, ranging from 0 (discordant) to 1 (highly concordant), were generated to assess the likelihood that a patient’s methylation profile matches a known disorder. A positive classification typically requires an MVP score >0.5, supported by concordant hierarchical clustering and multidimensional scaling patterns.

## Results

### Patient cohort

**FIGURE 1:**
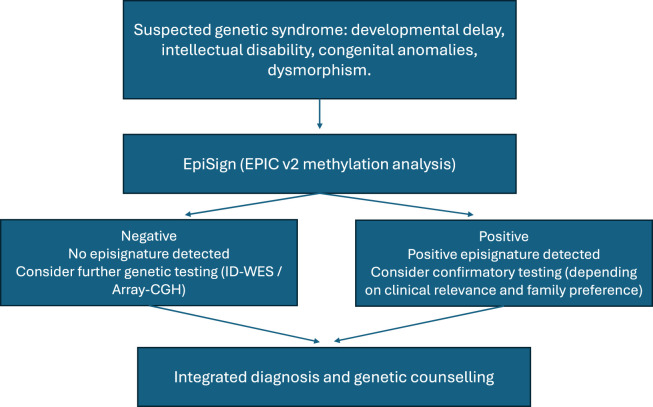
Flow diagram illustrating the diagnostic pathway of patients included in this study. Patients were selected based on clinical suspicion of a rare genetic disorder and underwent EpiSign™ episignature analysis as part of the diagnostic workflow. Where clinically indicated, positive episignature findings were followed by confirmatory molecular testing (ID-WES and/or CGH-array), depending on clinical relevance, potential consequences for patient management, and family preferences.

The study included 19 patients from the Dutch Caribbean (13 males and 6 females), with ages ranging from 2 to 25 years (Table 1). Patients were selected based on high clinical suspicion of a syndromic genetic syndrome, defined as the presence of developmental delay and/or intellectual disability, in combination with congenital anomalies, dysmorphic features, or other clinical findings suggestive of a genetic syndrome (Figure 1).

**TABLE 1:** Patient cohort.

Patient	Gender	Age in years	Episignature	Final diagnosis	Molecular test
1	Male	11	None	​	arr(X,Y)x1,(1–22)x2
2	Male	5	Fragile X	Fragile X syndrome	arr(X,Y)x1,(1–22)x2, positive *FMR1* CGG-repeat test
3	Female	14	None	​	arr(X,1–22)x2
4	Male	15	None	​	-
5	Male	3	Down syndrome	Down syndrome	arr(21)x3
6	Male	3	Cornelia de Lange syndrome	​	ID-WES: *PUF60*: c.688del p.(Val230Trpfs*58)
7	Female	12	None	​	-
8	Male	13	None	​	-
9	Male	8	MRX93 BRWD3	X-linked intellectual developmental disorder-93	ID-WES: *BRWD3*: c.3863del, p.(Lys1288Argfs*11)
10	Female	6	None	​	arr(X,1–22)x2
11	Male	2	None	​	arr(1–22)x2,(X,Y)x1, ID-WES: *EP300*: c.5332G>T, p.(Gly1778Trp) (VUS) Does not map to any of the known episignature disorders
12	Male	17	None	​	arr(1–22)x2,(X,Y)x1, ID-WES: *ZSWIM6*: c.1323G>A, p.(Trp441*) (VUS), *ANKRD11*: c.6552_6557del, p.(Glu2185_Glu2186del) (VUS), *ASXL1*: c.1766C>G, p.(Pro589Arg) (VUS) *TNRC6B*: c.4152G>A, p.(Met1384Ile) (VUS), *HCFC1*: c.4376C>T, p.(Thr1459Ile) (VUS) Does not map to any of the known episignature disorders
13	Female	25	None	​	-
14	Male	8	None	​	arr(1–22)x2,(X,Y)x1, ID-WES: normal
15	Male	9	ReNU syndrome	ReNU syndrome	Sanger: *RNU4-2*:n.64_65insT
16	Female	25	Kleefstra syndrome 1	Kleefstra syndrome 1	ID-WES: *EHMT1*: c.2807A>T, p.(Asn936Ile) (VUS)
17	Male	14	None	​	ID-WES: *TRRAP*: c.11487G>A, p.(Trp3829*) (VUS) Does not map to any of the known episignature disorders
18	Male	7	None	​	ID-WES: normal
19	Male	8	None	​	ID-WES: normal

### EpiSign diagnostic yield

**FIGURE 2:**
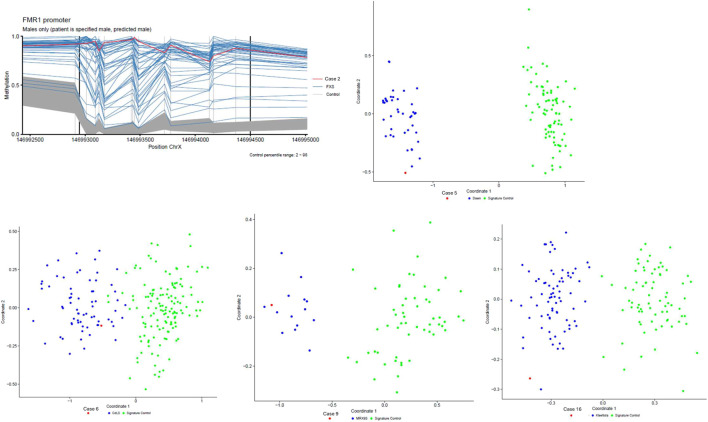
EpiSign™ MDS plots and FMR1 promoter analysis. Multidimensional scaling (MDS) plots showing episignature-based classification of four representative patients compared to reference control and disease-specific cohorts. Each sample is displayed relative to established episignature clusters, with classification based on clustering proximity. In addition, targeted analysis of the FMR1 promoter region is shown for the patient with suspected Fragile X syndrome. The case that tested positive for ReNU syndrome is not included in this figure because the finding was obtained in a research setting.

EpiSign™ identified a positive episignature in six patients (32% of the cohort). Diagnoses included Fragile X syndrome (case 2), Down syndrome (case 5), Cornelia de Lange syndrome (case 6), Intellectual Developmental Disorder, X-linked 93 (MRX93, case 9), ReNU syndrome (case 15), and Kleefstra syndrome 1 (case 16). Positive findings were validated or further characterized by targeted molecular analyses where clinically indicated. Although Down syndrome can be diagnosed clinically and confirmed by karyotyping, the corresponding episignature was included as an internal validation of the EpiSign™ platform and demonstrated correct classification of a well-established chromosomal methylation signature. A representative episignature plot is shown for most cases; however, no MDS visualization is included for case 15 (ReNU syndrome), as this case was analyzed in a research-only context outside the standardized diagnostic reporting workflow (Figure 2).

### Case summaries

- Case 2: Fragile X syndrome was confirmed by the presence of a CGG repeat expansion in the *FMR1* gene.
- Case 5: Down syndrome was identified by the characteristic episignature and via karyotyping (trisomy 21).
- Case 6: The Cornelia de Lange syndrome episignature was detected. ID-WES revealed a pathogenic variant in *PUF60* (c.688del, p.Val230Trpfs*58), which has potential links to Cornelia de Lange-related genes (Hoogenboom et al., 2024). However, this might also be a false positive call because of episignature overlap. To date, no episignature has been defined for Verheij syndrome due to the limited availability of affected patients for reference cohort development.
- Case 9: The Intellectual Developmental Disorder X-linked 93 (MRX93) episignature was present, and variants in *BRWD3* (c.3863del, p.Lys1288Argfs*11) were confirmed by ID-WES.
- Case 15: The ReNU syndrome episignature was positive and confirmed by Sanger sequencing.
- Case 16: The Kleefstra syndrome 1 episignature was positive and supported by a variant in *EHMT1* (c.2807A>T, p.Asn936Ile, VUS) detected by ID-WES.

### Variants of uncertain significance (VUS) and negative cases

Several patients without positive episignatures harbored VUS identified via ID-WES.

- Case 11 had a VUS in EP300, associated with Rubinstein-Taybi syndrome 1 and 2.
- Case 12 presented multiple VUS in genes including *ZSWIM6*, *ANKRD11*, *ASXL1*, *TNRC6B*, and *HCFC1*, none of which overlapped with known episignatures.

Other patients were negative both for known episignatures and for pathogenic variants (e.g., cases 13, 14, 18, and 19), highlighting that some disorders may involve genes or epigenetic patterns not yet represented in the EpiSign™ database.

### Summary of findings

Overall, EpiSign™ identified a clinically informative episignature in 32% of the cohort (6/19), representing a preliminary estimate of its diagnostic utility in this resource-limited setting. Cases with VUS emphasize the potential for future reanalysis as new episignatures are incorporated into the database.

## Discussion

In this study, we evaluated EpiSign™ as a first-tier diagnostic tool in 19 patients from the Dutch Caribbean and confirmed positive findings using ID-WES and/or CGH-array. While advanced genomic testing such as WES remains the gold standard due to higher diagnostic yield, practical limitations in the Dutch Caribbean (logistics, cost, and lack of parental samples) necessitate a modified diagnostic workflow. While EpiSign™ was modelled as a first-tier test for analytical purposes, in practice it often serves as a complementary tool to standard genomic testing. It is particularly valuable for interpreting VUS when parental samples are unavailable and can help guide targeted follow-up testing, highlighting its potential utility in resource-limited settings. These results are consistent with recent findings by Tkemladze et al. (2025), who demonstrated that DNA methylation arrays could achieve a 30.6% diagnostic yield as a first-tier test in individuals with suspected genetic syndromes, particularly for syndromic and imprinting disorders, supporting the utility of episignature-based screening in selected patient populations (Tkemladze et al., 2025).

Six patients (32%) received a definitive diagnosis through EpiSign™, although one case (Down syndrome) could be clinically identified and quickly diagnosed through standard cytogenetic analysis. The diagnostic yield is reported descriptively and should be interpreted as a preliminary estimate given the limited sample size. Notably, positive episignatures were confirmed by independent molecular analyses, including targeted sequencing, and ID-WES. It should be noted that episignature overlap is generally limited; however, certain disorders belong to broader epigenetic “umbrella” signatures, where related genes or syndromic entities may cluster within shared methylation domains rather than representing true false-positive results.

Long-read sequencing was not incorporated in the current workflow, as this study focused on episignature-based diagnostics in a resource-limited setting. While increasingly used for detecting structural variants and repeat expansions, its implementation in routine diagnostics is still evolving and not yet widely available in all clinical settings (van der Laan et al., 2026; Smits et al., 2026). Nevertheless, it represents a valuable complementary approach for future studies and is already being adopted by several groups to improve diagnostic yield in rare disease cohorts.

Importantly, as a first-tier test, episignature analysis does not identify the underlying genetic defect. Nonetheless, in many clinical scenarios, this classification is sufficient to guide patient management and inform family counselling. In some cases, the primary diagnostic question is to establish a clinical diagnosis rather than to determine recurrence risk or enable prenatal testing, particularly in settings where such options are limited or not part of the immediate clinical decision-making process. In these situations, episignature analysis can provide actionable information without requiring parental samples or extensive sequencing infrastructure. However, interpretation may be complicated by potential false positives, and confirmation in the identified gene remains recommended when clinically relevant.

Another key strength of episignature testing is its dynamic reference database: previously tested samples are automatically reanalyzed as new episignatures are discovered. This means that patients initially classified as negative (based on the current classifier) may later receive a diagnosis without additional testing or cost, enhancing the long-term clinical value of the assay. In VUS cases (e.g., Cases 11 and 12), episignature analysis provided additional interpretative context; in Case 12, the absence of a matching episignature across multiple candidate variants supported prioritization away from known episignature-associated syndromes.

It should be emphasized that episignature testing is not suitable for prenatal diagnostics and not recommended in the neonatal period, due to dynamic changes in the epigenome during early development. Its clinical utility is therefore greatest in children older than 1 year and adults presenting with neurodevelopmental phenotypes, where methylation patterns are stable and interpretable.

The EpiSign™ reference database is composed of a large and diverse set of control samples representing multiple ancestries, age groups, and clinical backgrounds. Classification is based on heterogeneous reference cohorts with adjustment for key covariates such as age and sex. While no population-specific Dutch Caribbean control cohort is available as yet, the use of diverse reference data supports generalizability of the classification framework, although residual population-related variability cannot be fully excluded.

Beyond diagnostic utility, inclusion of patients from the Dutch Caribbean increases the ethnic diversity of reference cohorts and may help reduce population bias in episignature-based classification. For example, a patient with a 6p22.3 deletion involving *JARID2* (case 1) supported the development of the *JARID2* episignature (Verberne et al., 2022b). Similarly, twin brothers carrying a likely pathogenic variant in *USP7* contributed to the characterization of Hao-Fountain syndrome (van der Laan et al., 2023). These contributions underline the importance of including underrepresented populations in reference cohorts, which can reduce uncertainty in VUS interpretation and improve diagnostic and healthcare equity, without necessarily increasing the number of pathogenic diagnoses. Those cases were not included in this study as they represent previously published patients with a confirmed molecular diagnosis who contributed to earlier episignature discovery efforts rather than to the current diagnostic evaluation.

Overall, our findings demonstrate that EpiSign™ can function as a supportive first-line tool in settings with limited diagnostic infrastructure, helping to guide further testing when sequencing or parental analyses are delayed or unavailable.

## Conclusion

EpiSign™ is a rapid and adaptable diagnostic method that could serve as a first-tier tool when modelled in the workflow, providing complementary insights to standard testing for patients in the Dutch Caribbean. It identified specific disorders in 32% of the cohort (6/19), with confirmation by independent molecular analyses. This represents a preliminary estimate of diagnostic performance in a small cohort. The ability to automatically reanalyze samples as new episignatures are discovered further increases its diagnostic potential over time. This approach is particularly relevant in regions with limited access to advanced genetic testing, parental samples and prenatal diagnostics. Although EpiSign™ can establish a diagnosis with high confidence, it does not identify the underlying genetic defect. Nevertheless, obtaining a confirmed clinical classification can guide more targeted follow-up sequencing once resources permit. Inclusion of diverse populations in episignature reference cohorts enhances global diagnostic capabilities and promotes equitable access to molecular diagnosis for rare disorders.

## Data Availability

The original contributions presented in the study are included in the article/supplementary material. Individual genomic, epigenomic, and other potentially identifiable data contained within the EpiSign Knowledge Database (EKD) cannot be deposited in publicly accessible repositories due to institutional and ethical restrictions. These include: (i) data and samples submitted from external institutions to London Health Sciences Centre under Institutional Material and Data Transfer Agreements; (ii) data submitted for episignature assessment under Research Services Agreements; and (iii) research cohorts governed by Institutional Research Ethics Board approvals (Western University REB 106302 and REB 116108). Some of the software tools used in this study are publicly available as described in the Materials and Methods. EpiSign™ is a commercial software platform and is not publicly available. Further inquiries can be directed to the corresponding author.

## References

[B2] Aref-Eshghi E. Kerkhof J. Pedro V. P. Barat-Houari M. Ruiz-Pallares N. Andrau J. C. (2020). Evaluation of DNA methylation episignatures for diagnosis and phenotype correlations in 42 mendelian neurodevelopmental disorders. Am. J. Hum. Genet. 106 (3), 356–370. 10.1016/j.ajhg.2020.01.019 32109418 PMC7058829

[B3] Hoogenboom A. Falix F. A. van der Laan L. Kerkhof J. Alders M. Sadikovic B. (2024). Novel PUF60 variant suggesting an interaction between Verheij and Cornelia de Lange syndrome: phenotype description and review of the literature. Eur. J. Hum. Genet. 32, 435–439. 10.1038/s41431-023-01527-1 38273166 PMC10999433

[B4] Kerkhof J. Rastin C. Levy M. A. Relator R. McConkey H. Demain L. (2024). Diagnostic utility and reporting recommendations for clinical DNA methylation episignature testing in genetically undiagnosed rare diseases. Genet. Med. 26 (5), 101075. 10.1016/j.gim.2024.101075 38251460

[B5] Levy M. A. McConkey H. Kerkhof J. Barat-Houari M. Bargiacchi S. Biamino E. (2022). Novel diagnostic DNA methylation episignatures expand and refine the epigenetic landscapes of Mendelian disorders. HGG Adv. 3 (1), 100075. 10.1016/j.xhgg.2021.100075 35047860 PMC8756545

[B6] Sadikovic B. Aref-Eshghi E. Levy M. A. Rodenhiser D. (2019). DNA methylation signatures in mendelian developmental disorders as a diagnostic bridge between genotype and phenotype. Epigenomics 11 (5), 563–575. 10.2217/epi-2018-0192 30875234

[B7] Smits D. J. Ferraro F. Drost M. van der Linde H. C. de Graaf B. M. van Bever Y. (2026). Nanopore long-read sequencing for the critically ill facilitates ultrarapid diagnostics and urgent clinical decision making. Eur. J. Hum. Genet. 34, 108–118. 10.1038/s41431-025-01959-x 41116046 PMC12816666

[B8] Tkemladze T. Campbell C. Bregvadze K. Kvaratskhelia E. Abzianidze E. Demain L. (2025). Evaluating DNA methylation episignatures as a first-tier diagnostic test in individuals with suspected genetic disorders. Eur. J. Hum. Genet. 34, 296–299. 10.1038/s41431-025-01939-1 41044236 PMC12858995

[B9] van der Laan L. Karimi K. Rooney K. Lauffer P. McConkey H. Caro P. (2023). DNA methylation episignature, extension of the clinical features and comparative epigenomic profiling of Hao-Fountain syndrome caused by variants in USP7. Genet. Med. 26 (3), 101050. 10.1016/j.gim.2023.101050 38126281

[B10] van der Laan L. Haagmans M. A. Venema A. Kerkhof J. Levy M. A. Briuglia S. (2026). Nanopore sequencing enables combined detection of USP7 variants and a known Hao-Fountain syndrome episignature. Front. Genet. 16, 16–2025. 10.3389/fgene.2025.1730165 41555921 PMC12812390

[B11] Verberne E. A. Ecury-Goossen G. M. Manshande M. E. Ponson-Wever M. de Vroomen M. Tilanus M. (2021). Clinical and community genetics services in the Dutch Caribbean. J. Community Genet. 12 (3), 497–501. 10.1007/s12687-021-00515-6 33751485 PMC7943254

[B12] Verberne E. A. Westermann J. M. de Vries T. I. Ecury-Goossen G. M. Lo ANSM Manshande M. E. (2022a). Genetic care in geographically isolated small island communities: 8 years of experience in the Dutch Caribbean. Am. J. Med. Genet. A 188 (6), 1777–1791. 10.1002/ajmg.a.62708 35253369 PMC9314971

[B13] Verberne E. A. van der Laan L. Haghshenas S. Rooney K. Levy M. A. Alders M. (2022b). DNA methylation signature for JARID2-Neurodevelopmental syndrome. Int. J. Mol. Sci. 23 (14), 8001. 10.3390/ijms23148001 35887345 PMC9322505

